# Infections of respiratory or abdominal origin in ICU patients: what are the differences?

**DOI:** 10.1186/cc8909

**Published:** 2010-03-15

**Authors:** Elena Volakli, Claudia Spies, Argyris Michalopoulos, AB Johan Groeneveld, Yasser Sakr, Jean-Louis Vincent

**Affiliations:** 1 Dept of Intensive Care, Erasme Hospital, Université Libre de Bruxelles, Route de lennik 808, 1070 Brussels, Belgium; 2 Dept of Anesthesiology and Intensive Care Medicine, Campus Virchow-Klinikum and Campus Charité Mitte, Hindenburgdamm 30, D-12200 Berlin, Germany; 3 Intensive Care Unit, Henry Dunant Hospital, Department of Medicine, 107 Mesogion Av, 115 26 Athens, Greece; 4 Dept of Intensive Care, Institute for Cardiovascular Research, VU University Medical Center, De Boelelaan 1117, 1081 Amsterdam, The Netherlands; 5 Dept of Anesthesiology and Intensive Care, Friedrich-Schiller University, Erlanger Allee 101, D-07747 Jena, Germany

## Abstract

**Introduction:**

There are few data related to the effects of different sources of infection on outcome. We used the Sepsis Occurrence in Acutely ill Patients (SOAP) database to investigate differences in the impact of respiratory tract and abdominal sites of infection on organ failure and survival.

**Methods:**

The SOAP study was a cohort, multicenter, observational study which included data from all adult patients admitted to one of 198 participating intensive care units (ICUs) from 24 European countries during the study period. In this substudy, patients were divided into two groups depending on whether, on admission, they had abdominal infection but no respiratory infection or respiratory infection but no abdominal infection. The two groups were compared with respect to patient and infection-related characteristics, organ failure patterns, and outcomes.

**Results:**

Of the 3,147 patients in the SOAP database, 777 (25%) patients had sepsis on ICU admission; 162 (21%) had abdominal infection without concurrent respiratory infection and 380 (49%) had respiratory infection without concurrent abdominal infection. Age, sex, and severity scores were similar in the two groups. On admission, septic shock was more common in patients with abdominal infection (40.1% vs. 29.5%, *P *= 0.016) who were also more likely to have early coagulation failure (17.3% vs. 9.5%, *P *= 0.01) and acute renal failure (38.3% vs. 29.5%, *P *= 0.045). In contrast, patients with respiratory infection were more likely to have early neurological failure (30.5% vs. 9.9%, *P *< 0.001). The median length of ICU stay was the same in the two groups, but the median length of hospital stay was longer in patients with abdominal than in those with respiratory infection (27 vs. 20 days, *P *= 0.02). ICU (29%) and hospital (38%) mortality rates were identical in the two groups.

**Conclusions:**

There are important differences in patient profiles related to the site of infection; however, mortality rates in these two groups of patients are identical.

## Introduction

Infection is a major challenge in the intensive care unit (ICU). Cited prevalence rates of ICU infection vary between 45% to 58% [1,2], and incidence rates between 30% to 35% [3,4]. Infections are already present on admission to the ICU in about 50% of cases; rates are perhaps even higher in studies limited to critically ill patients [1-6].

It has been shown that infections originating from the urinary tract usually have a better outcome than infections from other sources [7-10]. However, whether there are differences in outcomes for other sources of sepsis is not well defined. Lung and abdominal infections are the most common infections in the ICU [3,4,6,11], and several studies have suggested that, although respiratory infections are more common, abdominal infections may be more severe [3,10,12-15]. However, whether this translates into worse outcomes is unclear. Importantly, if outcomes vary according to the source of infection, this may impact on clinical trial design, as currently patients with infections from different sources are often grouped together.

The aim of the present study was, therefore, to investigate whether the presence at ICU admission of infections originating in these two sites, abdomen and lung, had any impact on patterns of organ failure or on patient outcome. For this purpose, we used the database of the Sepsis Occurrence in Acutely Ill Patients (SOAP) study [6], a large systematic cohort study performed in European ICU patients.

## Materials and methods

### Study design

The SOAP study was a prospective multicenter observational study designed to evaluate the epidemiology and characteristics of sepsis in European countries and was initiated by a working group of the European Society of Intensive Care Medicine. Full details of recruitment, data collection and management have been provided elsewhere [6]. Briefly, all adult patients (> 15 years old) admitted to a participating center (see Additional file 1 for a list of participating countries and centers) between 1 and 15 May 2002 were included, except patients who stayed in the ICU for less than 24 hours for routine postoperative surveillance. Due to the observational character of the study which did not require any deviation from routine medical care, institutional review board approval was either waived or expedited in participating institutions and informed consent was not required. Patients were followed up until death, hospital discharge, or for 60 days.

### Data collection and management

Data were collected prospectively using pre-printed case report forms and entered centrally by medical personnel. Data collection on ICU admission included demographic data, comorbid diseases, admission category, source of admission and admission diagnosis. Clinical and laboratory data needed to calculate the Simplified Acute Physiology Score II (SAPS II) were reported as the worst value within 24 hours after hospital admission [16]. Evaluation of organ function was made using the Sequential Organ Failure Assessment (SOFA) score, based on the most abnormal value for each of the six organ systems [17]. Daily collection of data included infection characteristics, organ function and the need for special supportive modalities such as mechanical ventilation, hemofiltration and hemodialysis.

### Definitions

Infection was defined as the presence of a pathogenic microorganism in a sterile milieu and/or clinically suspected infection, plus the administration of antibiotics. Clinically suspected infection was diagnosed at the discretion of the attending physician. Sepsis and severe sepsis and septic shock were defined by standard criteria [18]. Organ failure was defined as a Sequential Organ Failure Assessment (SOFA) score > 2 for the organ in question [17]. Early organ failure and late organ failure were defined as those occurring within and after 48 hours of a diagnosis of sepsis, respectively. For the purposes of this substudy, two groups were identified: Patients with abdominal infection (microbiologically proven or clinical diagnosis) on admission to the ICU without any concurrent respiratory infection and those with respiratory infections (microbiologically proven or clinical diagnosis) on ICU admission without concurrent abdominal infection. Secondary infections were defined as infections occurring more than 24 hours after onset of a preexisting infection, at a site other than the abdominal or respiratory system for patients in the abdominal or respiratory groups, respectively.

### Statistical analysis

Data were analyzed using the Statistical Package for Social Sciences (SPSS) for Windows, version 17.0 (SPSS Inc., Chicago, IL, USA). A Kolmogorov-Smirnov test was used, and histograms and normal-quantile plots were examined to verify the normality of distribution of continuous variables. Discrete variables are expressed as counts (percentage) and continuous variables as means ± SD or median (25^th ^to 75^th ^percentiles). For demographic and clinical characteristics of the study groups, differences between groups were assessed using a chi-square, Fisher's exact test, Student's t-test or Mann-Whitney U test, as appropriate. We performed a multivariate logistic regression analysis with development of secondary infection as the dependent factor to investigate the influence of length of ICU stay on the development of secondary infection in abdominal and respiratory groups. Variables considered for the analysis included, demographic data, co-morbidities, SAPS II score on admission, type of microorganism, organ failure assessed by the SOFA score. Only variables associated with a higher risk of development of secondary infection (*P *< 0.2) on a univariate basis were modeled. All variables included in the model were tested for colinearity. Interaction terms involving combinations between length of ICU stay and presence in the abdominal or respiratory group were tested. A Hosmer and Lemeshow goodness of fit test was performed and odds ratios and their corresponding 95% confidence intervals were calculated [19]. We also performed a multivariate Cox proportional hazard model with time to in-hospital death as the dependent factor. Variables included in the Cox regression analysis were: age, gender, comorbid diseases, SAPS II and SOFA scores on admission, the type of admission (medical or surgical), source of admission, admission diagnosis, the presence of sepsis, early organ failure, and the need for mechanical ventilation or renal replacement therapy during the ICU stay. Variables were introduced in the model if significantly associated with a higher risk of in-hospital death on a univariate basis at a *P*-value < 0.2. Colinearity between variables was excluded prior to modeling. The time dependent covariate method was used to check the proportional hazard assumption of the model; an extended Cox model was constructed, adding interaction terms that involve time, that is, time dependent variables, computed as the by-product of time and individual covariates in the model (time × covariate). Individual time-dependent covariates were introduced one by one and in combinations in the extended model, none of which was found to be significant. A stepwise approach was used and presence in the abdominal or respiratory group variable was forced as the last step in the model. A Kaplan-Meier survival analysis was performed and survival between groups was compared using a Log rank Test. All statistics were two-tailed and a *P *< 0.05 was considered to be statistically significant.

## Results

### Study population

Of the 3,147 patients enrolled in the SOAP study, 777 (25%) had sepsis on admission to the ICU; of these, 162 (21%) had abdominal infection without concurrent respiratory infection and 380 (49%) had respiratory infection without concurrent abdominal infection. The baseline characteristics of the patients are summarized in Table 1. Age, sex, SAPS II and SOFA scores were similar in the two groups. Patients with abdominal infections were more likely to be surgical admissions and to have been referred from the operating room or recovery room; they were more likely than patients with respiratory infections to have cancer but less likely to have chronic obstructive pulmonary disease (COPD) or hematologic cancer. Patients with respiratory infection were admitted mainly because of respiratory (57%), cardiovascular (19%) and neurologic diagnoses (13%), while patients with abdominal infection were primarily admitted because of digestive/liver (40%) and cardiovascular diagnoses (34%).

**Table 1:** Baseline characteristics and outcomes

Characteristic	All patients (n = 542)	Abdominal infection (n = 162)	Respiratory infection (n = 380)	*P*-value
Age, years	63.2 ± 15.7	65.1 ± 15.0	62.4 ± 16.0	0.11
Male	314 (58.4%)	89 (55.3%)	225 (59.7%)	0.34
SAPS II score	43.6 ± 17.1	43.1 ± 17.7	43.9 ± 16.8	0.42
SOFA score	6.5 ± 4.1	6.4 ± 4.0	6.6 ± 4.2	0.65
Co-morbidities				
Cancer	79 (14.6%)	34 (21.0%)	45 (11.8%)	0.006
Hematologic cancer	26 (4.8%)	2 (1.2%)	24 (6.3%)	0.01
COPD	107 (19.7%)	19 (11.7%)	88 (23.2%)	0.002
Cirrhosis	26 (4.8%)	10 (6.2%)	16 (4.2%)	0.32
HIV and/or AIDS	7 (1.3%)	0	7 (1.8%)	0.10
Heart failure	42 (7.7%)	8 (4.9%)	34 (8.9%)	0.11
Diabetes	35 (6.5%)	9 (5.6%)	26 (6.8%)	0.57
Admission category				
Medical	333 (61.4%)	32 (19.8%)	301 (79.2%)	< 0.001
Surgical	209 (38.6%)	130 (80.2%)	79 (20.8%)	< 0.001
Elective	82 (15.1%)	35 (21.6%)	47 (12.4%)	
Emergency	127 (23.4%)	95 (58.6%)	32 (8.4%)	
Source of admission				< 0.001
ER/Ambulance	118 (24.0%)	17 (11.6%)	101 (29.3%)	
Hospital floor	191 (38.9%)	40 (27.4%)	151 (43.8%)	
OR/Recovery	126 (25.7%)	82 (56.2%)	44 (12.8%)	
Hospital other	56 (11.4%)	7 (4.8%)	49 (14.2%)	
Admission diagnosis				< 0.001
Monitoring	15 (2.8%)	7 (4.5%)	8 (2.1%)	
Neurologic	51 (9.5%)	3 (1.9%)	48 (12.7%)	
Respiratory	229 (42.8%)	15 (9.6%)	214 (56.6%)	
Cardiovascular	123 (23.0%)	53 (33.8%)	70 (18.5%)	
Renal	14 (2.6%)	9 (5.7%)	5 (1.3%)	
Digestive/liver	73 (13.6%)	63 (40.1%)	10 (2.6%)	
Trauma	16 (3.0%)	4 (2.5%)	12 (3.2%)	
Others	14 (2.7%)	3 (2.0%)	11 (2.9%)	
Sepsis syndromes				
Severe sepsis	391 (72.1%)	113 (69.8%)	278 (73.2%)	0.41
Septic shock	177 (32.7%)	65 (40.1%)	112 (29.5%)	0.01
Length of ICU stay (days)	6 (3 - 13)	6 (2 - 15)	6 (3 - 13)	0.95
Length of hospital stay (days)	21 (10 - 44)	27 (13 - 48)	20 (10 - 41)	0.02
ICU mortality	157 (29.0%)	47 (29.0%)	110 (28.9%)	0.98
Hospital mortality	204 (37.6%)	60 (37.5%)	144 (38.1%)	0.89

Data are expressed as mean ± standard deviation, number (percentage), or median (interquartile range). AIDS: acquired immune deficiency syndrome; COPD: chronic obstructive pulmonary disease; ER: emergency room; HIV: human immunodeficiency virus; ICU: intensive care unit; OR: operating room; SAPS II: Simplified Acute Physiology Score; SOFA: Sequential Organ Failure Assessment

### Infection-related characteristics

Table 2 shows the major microbiological data. Microbiologic cultures were positive in 46% of the patients. Diagnostic criteria for infection and the overall rates of Gram-positive, Gram-negative, or fungal infection were similar in the two groups. The most commonly isolated organisms in patients with abdominal infections were *Staphylococcus aureus *and *Streptococcus *group D, and in patients with respiratory infections, the most commonly isolated organisms were *S. aureus *and *Pseudomonas *species. *Streptococcus pneumoniae *infections were more common in patients with respiratory than in those with abdominal infections (4.7% vs. 0.6%, *P *= 0.02), while *Streptococcus *group D (18.5% vs. 6.3%, *P *< 0.001) and any streptococcal (24.1% vs. 12.9%, *P *< 0.001) infections were more common in patients with abdominal infections. *Escherichia coli *(15.4% vs. 7.6%, *P *= 0.006) and *Candida *non-albicans (6.2% vs. 2.4%, *P *= 0.027) infections were also more common in patients with abdominal infections than in those with respiratory infections.

**Table 2:** Diagnostic criteria for infection and the microorganisms isolated in patients with abdominal and respiratory infections

Characteristic	All patients (n = 542)	Abdominal infection (n = 162)	Respiratory infection (n = 380)	*P*-value
Diagnostic criteria				
Isolates only	44 (8.1%)	9 (5.6%)	35 (9.2%)	0.17
Clinical only	294 (54.2%)	91 (56.2%)	203 (53.4%)	0.57
Both	204 (37.6%)	62 (38.3%)	142 (37.4%)	0.85
Class/microorganism				
Gram-positive	130 (23.9%)	42 (25.9%)	88 (23.2%)	0.58
Gram-negative	144 (26.5%)	43 (26.5%)	101 (26.6%)	0.64
Anaerobes	9 (1.6%)	7 (4.3%)	2 (0.5%)	0.45
Atypical microorganisms	4 (0.7%)	0	4 (1.1%)	0.323
Fungi	81 (14.9%)	30 (18.5%)	51 (13.4%)	0.148
Gram-positive				
Any *Staphylococcus*	119 (22.0%)	36 (22.2%)	83 (21.8%)	0.92
*Staphylococcus aureus*	91 (16.8%)	30 (18.5%)	61 (16.1%)	0.48
MRSA	59 (10.9%)	22 (13.6%)	37 (9.7%)	0.18
*Staphylococcus*, others	70 (12.9%)	19 (11.7%)	51 (13.4%)	0.59
Any *Streptococcus*	88 (16.2%)	39 (24.1%)	49 (12.9%)	< 0.001
*Streptococcus *group D	54 (10.0%)	30 (18.5%)	24 (6.3%)	< 0.001
*Streptococcus pneumoniae*	19 (3.5%)	1 (0.6%)	18 (4.7%)	0.02
*Streptococcus*, others	19 (3.5%)	9 (5.6%)	10 (2.6%)	0.09
Gram-positive bacilli	15 (2.8%)	3 (1.9%)	12 (3.2%)	0.57
Gram-positive, others	10 (1.8%)	1 (0.6%)	9 (2.4%)	0.29
Gram-negative				
*Pseudomonas *species	67 (12.4%)	19 (11.7%)	48 (12.6%)	0.77
*Escherichia coli*	54 (10.0%)	25 (15.4%)	29 (7.6%)	0.006
*Enterobacter*	25 (4.6%)	11 (6.8%)	14 (3.7%)	0.11
*Klebsiella*	25 (4.6%)	6 (3.7%)	19 (5.0%)	0.51
*Proteus*	15 (2.8%)	6 (3.7%)	9 (2.4%)	0.38
*Acinetobacter*	17 (3.1%)	2 (1.2%)	15 (3.9%)	0.11
*Haemophilus*	12 (2.2%)	1 (0.6%)	11 (2.9%)	0.12
Gram-negative bacilli	36 (6.6%)	12 (7.4%)	24 (6.3%)	0.64
Gram-negative, others	90 (16.6%)	26 (16.0%)	64 (16.8)	0.82
Fungi				
*Candida albicans*	61 (11.3%)	21 (13.0%)	40 (10.5%)	0.41
*Candida*, others	19 (3.5%)	10 (6.2%)	9 (2.4%)	0.02
Fungi, others	7 (1.3%)	2 (1.2%)	5 (1.3%)	1
Viral/parasitic	9 (1.7%)	1 (0.6%)	8 (2.1%)	0.21

CSF: cerebrospinal fluid; MRSA: methicillin-resistant *S. aureus*; *Staphylococcus*, others includes methicillin-sensitive *S. aureus *and *Staphylococcus *coagulase negative methicillin-sensitive; *Streptococcus*, others includes *Streptococcus *A, B, C, G group and others; Gram-positive bacilli includes, *Moraxella *and others; Gram-negative, others includes *Salmonella, Serratia, Citrobacter, Stenotrophomonas maltophilia, Campylobacter*, other enterobacteroids, Gram-negative cocci; Anaerobes includes *Clostridium, Bacteroides*, anaerobic cocci, and others; Atypical microorganisms includes *Mycobacteria, Chlamydia, Rickettsia, Legionella pneumonia; *Fungi, others includes *Aspergillus *and others. The microorganism was considered once per patient even if present in more than one site.

**Figure 1:**
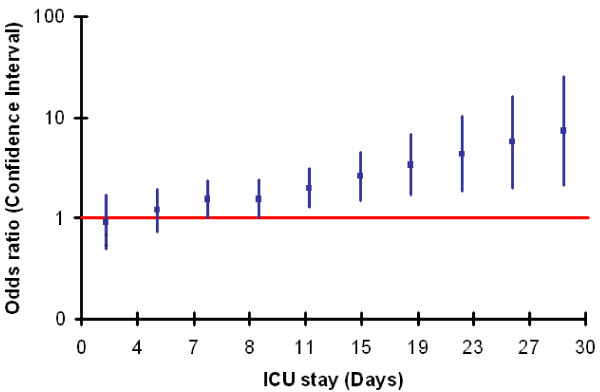
**The odds ratios of developing secondary infection in the abdominal group for different durations of ICU stay**. The solid line represents the point of significance; ICU stays longer than seven days were associated with a significant risk of developing secondary infection.

Secondary infections were more common in patients with abdominal infections (70 patients, 43%), than in those with respiratory infections (119 patients, 31%), *P *= 0.010. Thirty-five patients (22%) with abdominal infections developed respiratory infections later during the ICU stay and 15 patients (4%) with respiratory infections developed abdominal infections (Table 3). Patients with abdominal infection on admission were more likely to develop secondary skin/wound infection (16% vs. 5.5%, *P *< 0.001) whereas patients with respiratory infections were more likely to develop secondary urinary infections (9.2% vs. 1.9%, *P *< 0.001). Patients in the abdominal group who developed secondary infections had a longer ICU stay than those who did not (12 (5.7 to 27.3) days versus 9.8 (4.6 to 21.9), *P *< 0.05). Multiple logistic regression analysis showed that the relationship between the abdominal group and the development of secondary infection was related to ICU stay (interaction parameter = 0.069, *P *= 0.011 (Table 4). Specifically, the odds ratio of developing secondary infections increased with increasing duration of ICU stay in the abdominal group (Figure 1).

**Table 3:** Type of secondary infections

	Abdominal infection	Respiratory infection	*P-* value
Respiratory	35 (21.6%)	NA	-
Abdominal	NA	15 (3.9%)	-
Skin/wound	26 (16.0%)	21 (5.5%)	< 0.001
Other	15 (9.3%)	27 (7.1%)	0.39
Unknown	4 (2.5%)	5 (1.3%)	0.46
Bloodstream	28 (17.3%)	48 (12.6%)	0.15
Urinary	3 (1.9%)	35 (9.2%)	< 0.001
Catheter	14 (8.6%)	20 (5.3%)	0.14
CSF	0	2 (0.5%)	1

NA: not applicable

**Table 4:** Multiple logistic regression analysis in patients with abdominal infections. The development of secondary infection was the dependent variable

	Estimated coefficient	SD	Odds ratio (95% CI)	*P*-value
SAPS II score, per point	0.016	0.006	1.016 (1.005 to 1.028)	0.005
ICU length of stay, per day	0.055	0.011	1.057 (1.034 to 1.079)	< 0.001
Abdominal/respiratory variable				
Respiratory infection	Reference			
Abdominal infection	-0.076	0.316	0.927 (0.499 to 1.721)	0.810
Abdominal/respiratory infection by ICU LOS	0.069	0.027	1.071 (1.016 to 1.129)	0.011

CI: confidence interval; SD: standard error of the estimate; Hosmer and Lemeshow Chi square 7.636, *P *= 0.470. The percentage of correct classifications is 71.4.

### Morbidity and mortality

Although the incidence of severe sepsis on admission was similar in the two groups (around 70%), more patients with abdominal infection had septic shock on admission than patients with respiratory infection (40.1% vs. 29.5%, *P *= 0.016). However, when considering the incidence of sepsis syndromes during the whole ICU stay, these differences lost statistical significance (Table 5).

**Table 5:** Organ dysfunction patterns

Characteristic	All patients (n = 542)	Abdominal infection (n = 162)	Respiratory infection (n = 380)	*P*-value
Sepsis syndromes at any time during the ICU stay				
Severe sepsis	449 (82.8%)	128 (79.0%)	321 (84.5%)	0.12
Septic shock	241 (44.5%)	81 (50.0%)	160 (42.1%)	0.09
Procedures during ICU stay				
Mechanical ventilation	437 (80.6%)	129 (79.6%)	308 (81.1%)	0.70
Hemofiltration	69 (12.7%)	29 (17.9%)	40 (10.5%)	0.02
Hemodialysis	27 (5.0%)	8 (4.9%)	19 (5.0%)	0.97
SOFA scores				
SOFA max	8.4 ± 4.4	8.4 ± 4.8	8.4 ± 4.3	0.90
SOFA mean	5.6 ± 3.9	5.6 ± 4.0	5.6 ± 3.9	0.95
Early organ failure^a^				
Renal	174 (32.1%)	62 (38.3%)	112 (29.5%)	0.04
Respiratory	286 (52.8%)	79 (48.8%)	207 (54.5%)	0.22
Coagulation	64 (11.8%)	28 (17.3%)	36 (9.5%)	0.01
Hepatic	33 (6.1%)	7 (4.3%)	26 (6.8%)	0.26
CNS	132 (24.4%)	16 (9.9%)	116 (30.5%)	< 0.001
Cardiovascular	249 (45.9%)	90 (55.6%)	159 (41.8%)	0.003
Late organ failure^b^				
Renal	74 (13.7%)	16 (9.9%)	58 (15.3%)	0.09
Respiratory	56 (10.3%)	17 (10.5%)	39 (10.3%)	0.93
Coagulation	16 (3.0%)	7 (4.3%)	9 (2.4%)	0.21
Hepatic	16 (3.0%)	8 (4.9%)	8 (2.1%)	0.07
CNS	27 (5.0%)	8 (4.9%)	19 (5.0%)	0.97
Cardiovascular	30 (5.5%)	6 (3.7%)	24 (6.3%)	0.22
Organ failure any time				
Renal	248 (45.8%)	78 (48.1%)	170 (44.7%)	0.46
Respiratory	342 (63.1%)	96 (59.3%)	246 (64.7%)	0.22
Coagulation	80 (14.8%)	35 (21.6%)	45 (11.8%)	0.003
Hepatic	49 (9.0%)	15 (9.3%)	34 (8.9%)	0.90
CNS	159 (29.3%)	24 (14.8%)	135 (35.5%)	< 0.001
Cardiovascular	279 (51.5%)	96 (59.3%)	183 (48.2%)	0.01

CNS: Central nervous system; ^a^, occurring within 48 hours of a diagnosis of sepsis;

^b^, occurring more than 48 hours after a diagnosis of sepsis

Patients with abdominal infection also had a greater incidence of early coagulation failure (17.3% vs. 9.5%, *P *= 0.01) and early acute renal failure (38.3% vs. 29.5%, *P *= 0.04), and more needed hemofiltration than patients with respiratory infection. Patients with respiratory infection were more likely to have early neurological failure than patients with abdominal infection (30.5% vs. 9.9%, *P *< 0.001).

**Figure 2:**
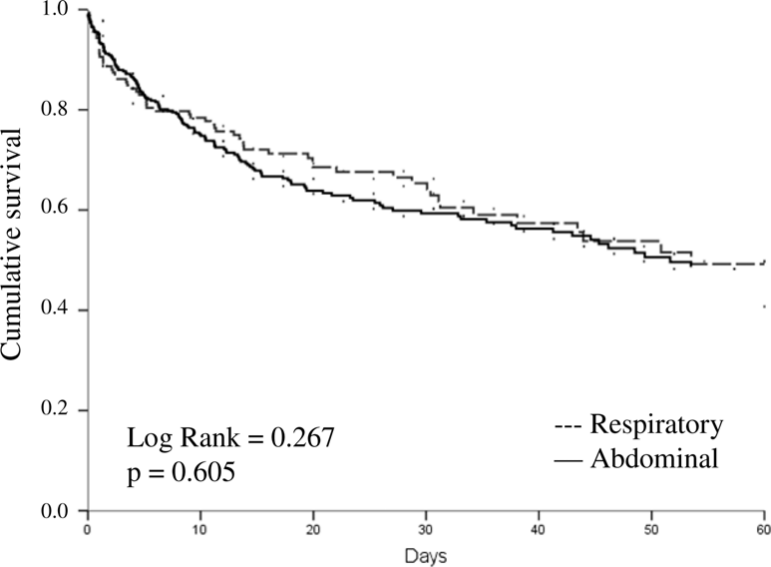
**Kaplan-Meier survival curves representing 60-day survival in patients with respiratory and those with abdominal infection**. Log Rank = 0.267: *P *= 0.605.

The median duration of ICU stay was the same in the two groups, but the median duration of hospital stay was longer for patients with abdominal infection (27 days vs. 20 days, *P *= 0.02). ICU (29.0% vs. 28.9%) and hospital (37.5% vs. 38.1%) mortality rates were remarkably similar in the two groups of patients. In a Kaplan Meier survival analysis, 60-day survival was similar between groups (Log Rank = 0.267, *P *= 0.605; Figure 2). In Cox regression analysis (Table 6), age, cancer, septic shock on admission, early coagulation failure, acute renal failure, and neurological failure were all associated with an increased risk of death, but abdominal or respiratory infection were not.

**Table 6:** Summary of Cox proportional hazard regression analysis with hospital mortality as the dependent variable

	Estimated coefficient	SE	Hazard ratio (95% CI)	*P*-value
Age, per year	0.04	0.01	1.04 (1.03 to 1.05)	< 0.001
Cancer	0.57	0.20	1.76 (1.20 to 2.59)	0.004
Septic shock on admission	0.42	0.15	1.52 (1.13 to 2.04)	0.006
Early coagulation failure	0.98	0.18	2.68 (1.88 to 3.80)	< 0.001
Early acute renal failure	0.6	0.15	1.83 (1.37 to 2.45)	< 0.001
Early neurological failure	0.36	0.16	1.43 (1.04 to 1.96)	0.029
Abdominal/respiratory variable				
Respiratory infection	reference			
Abdominal infection	0.28	0.19	1.32 (0.91 to 1.92)	0.149

CI: confidence interval; SE: standard error of the estimate

## Discussion

Using data from a large, prospective, pan-European database, we investigated the impact on organ failure and survival of the presence on admission of infection at two of the most common sites, the lung and the abdomen. On admission, patients with abdominal infection were more likely to have septic shock, early coagulation failure and early acute renal failure, and more needed hemofiltration than patients with respiratory infection. In contrast, patients with respiratory infections were more likely to have concurrent early neurological dysfunction than patients with abdominal infection. However, the median length of ICU stay was the same in the two groups and the two groups had identical ICU and hospital mortality rates.

The present study focused on infections originating from the lungs and the abdomen, because these two sites represent the most common causes of infection in acutely ill patients [3,4,6,11], and are also associated with higher workload and increased costs compared to other infections [20]. On admission, 49% of patients with sepsis had respiratory infections and 21% abdominal; overall in the SOAP study, 68% of patients had respiratory and 22% abdominal infections [6]. Similarly, in a study of 5,878 patients from Australia and New Zealand, the site of infection was pulmonary in 50% and abdominal in 19% of the episodes [11]. In another European study of 14,364 patients, the lung contributed to 62% of infections and intra-abdominal infections to 15% [3].

Although respiratory infections are more common, several studies have suggested that abdominal infections may be more severe [3,10,12-15]. The present study supports these findings, as more patients with abdominal infections than with respiratory infections had septic shock on admission. Nevertheless, mortality rates were similar in patients with abdominal and those with respiratory infections. The association between respiratory infection and a higher incidence of early neurological failure may be because respiratory infections are more common in patients with altered mental status or neurological diagnoses [21-23]; in our study, there was a higher proportion of neurological diagnoses in patients with respiratory infections than in those with abdominal infections. Moreover, although the assumed Glasgow coma score is supposed to be used for the SOFA score, it is possible that neurological dysfunction may have been overestimated in sedated patients. The association between abdominal infections and coagulation failure may be related to the fact that more patients with abdominal infections had septic shock, which frequently provokes coagulation abnormalities [24], or by the fact that most of these patients were postoperative, as surgery may be associated with altered coagulation [25,26]. However, all these suggestions remain speculative as our study design does not allow us to determine the reasons underlying these associations.

It has been fairly consistently reported that secondary infections are more frequent among patients who are already infected when admitted to the ICU, but differences in definitions make it difficult to compare studies [3,4,13,27,28]. Alberti et al. [3] reported that 26% of patients who were infected on ICU admission developed secondary infections compared to 15% of patients not infected on admission. Malacarne et al. [4] reported that 23% of patients admitted with infections developed secondary infections compared to 9% of those who were admitted without infection. Agarwal et al. reported that infection on admission was an independent risk factor for developing an ICU-acquired infection [27]. However, the above studies focused on patients admitted with any infection without distinguishing the type. In our study, secondary infections occurred more commonly in patients admitted with abdominal than with respiratory infection, related to their longer ICU stay as shown by the multivariate analysis. These patients also had a higher incidence of skin/wound infections compared to respiratory patients, likely related to more surgical wound infections. Merlino et al. [28], in a retrospective study of 168 patients with serious intra-abdominal infections, reported that 66 patients (40%) developed a secondary nosocomial infection. The presence of secondary infections is associated with an increased length of stay [29], but the effect of secondary infections on mortality is controversial, because patients who develop secondary infections are generally sicker and more likely to die [2-7,27,30].

Interestingly, there were no differences in ICU (29%) or hospital (38%) mortality between the two groups despite the greater incidence of septic shock on admission in patients with abdominal infections. Mortality rates in studies of infection and sepsis in the ICU are quite variable. In studies in surgical ICUs, ICU mortality rates in patients with abdominal infections varied from 22% to 72% [13-15,28,31-33]. ICU mortality rates for patients with community-acquired pneumonia range from 32% to 49% [22,34-36], and are perhaps higher in patients with hospital-acquired pneumonia [37]. Malacarne and colleagues found that among different sites of infection, only peritonitis diagnosed during the ICU stay was an independent prognostic factor for hospital mortality (OR 3.4, *P *= 0.0021) [4]. Although in our study, ICU lengths of stay were similar, the hospital length of stay was longer in patients with abdominal infection than in those with respiratory infection. We can speculate that this may be due to differences in baseline characteristics and the surgical nature of abdominal infections which can require more prolonged periods for resolution.

The advantage of our study is that it involves a large database from multiple centers with systematic collection of data. One limitation of the study is that the diagnoses of abdominal and respiratory infections were made at the discretion of the attending physician and criteria may have varied slightly from one center to another. As part of an observational study with a waiver of informed consent, we were unable to perform invasive tests to obtain more specific diagnoses and had to rely on what was routine clinical practice in the participating centers. In addition, we were unable to distinguish between hospital- and community-acquired infections. Moreover, septic shock was defined as the presence of infection plus the need for vasopressor agents, according to standard criteria at the time of the study. However, particularly in surgical patients, vasopressors may be required as a result of anesthetic agents, epidural anesthesia, blood loss, and so on, so that in the presence of infection it may be difficult to accurately distinguish the specific reason for vasopressor agents, thus confounding the diagnosis. Moreover, there were some differences in patient characteristics among the two groups of patients, but the multivariate analysis we performed adjusted for a large number of these and other variables which are known to influence outcome prediction.

## Conclusions

This analysis revealed that the two most common sources of infection on admission to the ICU are associated with different profiles. Patients with abdominal infection on admission are more likely to have septic shock on admission and to have early renal and coagulation failure, whereas patients with respiratory infection more commonly have early alteration in neurological function. The length of hospital stay in patients with abdominal infection is longer, likely because of the increased numbers of secondary infections in these patients. However, mortality rates were identical in the two groups of patients. These observations outline interesting differences depending on the source of sepsis, which may have important implications for our understanding of the epidemiology of sepsis and in the conduct of clinical trials.

## Key messages

• ICU patients admitted with abdominal infections have different profiles compared to those admitted with respiratory infections.

• ICU patients admitted with abdominal infections had longer hospital lengths of stay and increased numbers of secondary infections compared to patients admitted with respiratory infections.

• However, ICU and hospital mortality rates were the same regardless of the source of sepsis.

## Abbreviations

AIDS: acquired immunodeficiency syndrome; CNS: central nervous syndrome; COPD: chronic obstructive pulmonary disease; CSF: cerebrospinal fluid; ER: emergency room; ICU: intensive care unit; MRSA: methicillin-resistant *Staphylococcus aureus*; OR: operating room; SAPS: simplified acute physiology score; SOAP: Sepsis in Acutely ill Patients; SOFA: sequential organ failure assessment; SPSS: Statistical Package for SocialSciences.

## Competing interests

The authors declare that they have no competing interests.

## Authors' contributions

JLV conceived the initial SOAP study. EV, CS, AM, JG, YS and JLV participated in the design and coordination of the SOAP study. YS performed the statistical analyses. EV and JLV drafted the present manuscript. All authors read and approved the final manuscript.

## Supplementary Material

Additional file 1 **Participants by country (listed alphabetically)**. A Word file containing a list of participants by country, in alphabetical order. Click here for file
