# Supplementary material for: Infections of respiratory or abdominal origin in ICU patients: what are the differences?
Source: Crit Care. 2010 Mar 15;14(2):R32. doi: 10.1186/cc8909 (PMC2887138; doi:10.1186/cc8909)
Supplement: Additional file 1 — Participants by country (listed alphabetically). A Word file containing a list of participants by country, in alphabetical order. [file cc8909-S1.DOC]

**Additional file 1:** Participants by country (listed alphabetically)

*Austria:* University Hospital of Vienna (G. Delle Karth); LKH Steyr (V. Draxler); LKH-Deutschlandsberg (G. Filzwieser); Otto Wagner Spital of Vienna (W. Heindl); Krems of Donau (G. Kellner, T. Bauer); Barmherzige Bruede of Linz (K. Lenz); KH Floridsdorf of Vienna (E. Rossmann); University Hospital of Innsbruck (C. Wiedermann); *Belgium:* CHU of Charleroi (P. Biston); Hôpitaux Iris Sud of Brussels (D. Chochrad); Clinique Europe Site St Michel of Brussels (V. Collin); C.H.U. of Liège (P. Damas); University Hospital Ghent (J. Decruyenaere, E. Hoste); CHU Brugmann of Brussels (J. Devriendt); Centre Hospitalier Jolimont-Lobbes of Haine St Paul (B. Espeel); CHR Citadelle of Liege (V. Fraipont); UCL Mont-Godinne of Yvoir (E. Installe); ACZA Campus Stuivenberg (M. Malbrain); OLV Ziekenhuis Aalst (G. Nollet); RHMS Ath-Baudour-Tournai (J.C. Preiser); AZ St Augustinus of Wilrijk (J. Raemaekers); CHU Saint-Pierre of Brussels (A. Roman); Cliniques du Sud-Luxembourg of Arlon (M. Simon); Academic Hospital Vrije Universiteit Brussels (H. Spapen); AZ Sint-Blasius of Dendermonde (W. Swinnen); Clinique Notre-Dame of Tournai (F. Vallot); Erasme University Hospital of Brussels (J.L. Vincent); *Czech Republic:* University Hospital of Plzen (I. Chytra); U SV.Anny of Brno (L. Dadak); Klaudians of Mlada Boleslav (I. Herold); General Faculty Hospital of Prague (F. Polak); City Hospital of Ostrava (M. Sterba); *Denmark :* Gentofte Hospital, University of Copenhagen (M. Bestle); Rigshospitalet of Copenhagen (K. Espersen); Amager Hospital of Copenhagen (H. Guldager); Rigshospitalet, University of Copenhagen (K-L. Welling); *Finland:* Aland Central Hospital of Mariehamn (D. Nyman); Kuopio University Hospital (E. Ruokonen); Seinajoki Central Hospital (K. Saarinen); *France:* Raymond Poincare of Garches (D. Annane); Institut Gustave Roussy of Villejuif (P. Catogni); Jacques Monod of Le Havre (G. Colas); CH Victor Jousselin of Dreux (F. Coulomb); Hôpital St Joseph & St Luc of Lyon (R. Dorne); Saint Joseph of Paris (M. Garrouste); Hôpital Pasteur of Nice (C. Isetta); CHU Brabois of Vandoeuvre Les Nancy (J. Larché); Saint Louis of Paris (J-R. LeGall); CHU de Grenoble (H. Lessire); CHU Pontchaillou of Rennes (Y. Malledant); Hôpital des Hauts Clos of Troyes (P. Mateu); CHU of Amiens (M. Ossart); Hôpital Lariboisière of Paris (D. Payen); CHD Félix Gyuon of Saint Denis La Reunion (P. Schlossmacher); Hôpital Bichat of Paris (J-F. Timsit); Hôpital Saint Andre of Bordeaux (S. Winnock); Hôpital Victor Dupouy of Argentueil (J-P. Sollet); CH Auch (L. Mallet); CHU Nancy-Brabois of Vandoeuvre (P. Maurer); CH William Morey of Chalon (J-M. Sab); Victor Dupouy of Argenteuil (J-P. Sollet); *Germany:* University Hospital Heidelberg (G. Aykut); Friedrich Schiller University Jena (F. Brunkhorst); University Clinic Hamburg-Eppendorf (A. Nierhaus); University Hospital Mainz (M. Lauterbach); University Hospital Carl Gustav Carus of Dresden (M. Ragaller); Hans Sushemihl Krankenhaus of Emden (R. Gatz); Vivantes-Klinikum Neukoelln of Berlin (H. Gerlach); University Hospital RWTH Aachen (D. Henzler); Kreisklinik Langen-Seligenstadt (H-B Hopf**);** GKH Bonn (H. Hueneburg); Zentralklinik Bad Berka (W. Karzai); Neuwerk of Moenchengladbach (A. Keller); Philipps University of Marburg (U. Kuhlmann); University Hospital Regensburg (J. Langgartner); ZKH Links der Weser of Bremen (C. Manhold); University Hospital of Dresden (M. Ragaller); Universtiy of Wuerzburg (B. Reith); Hannover Medical School (T. Schuerholz); Universitätsklinikum Charité Campus Mitte of Berlin (C. Spies); Bethanien Hospital of Moers (R. Stögbauer); KhgmbH Schongau (J. Unterburger); *Greece:* Thriassio Hospital of Athens (P-M. Clouva-Molyvdas); Sismanoglion General Hospital of Athens (G. Giokas); KAT General Hospital of Athens (E. Ioannidou); G. Papanikolaou General Hospital of Thessaloniki (A. Lahana); Agios Demetrios of Thessaloniki (A. Liolios); Onassis Cardiac Surgery Center of Athens (K. Marathias); University Hospital of Ioannina (G. Nakos); Tzanio Hospital of Athens (A. Tasiou); Athens Gen. Hosp. Gennimatas (H. Tsangaris); *Hungary:* Peterfy Hospital of Budapest (P. Tamasi); *Ireland:* Mater Hospital of Dublin (B. Marsh); Beaumont Hospital of Dublin (M. Power); *Israel:* Hadassah Hebrew University Medical Center (C. Sprung); *Italy:* Azienda Ospedaliera Senese o Siena (B. Biagioli); S. Martino of Genova (F. Bobbio Pallavicini); Azienda Ospedaliera S. Gerardo dei Tintori of Monza (A. Pesenti); Osp Regionale of Saronno (C. Capra); Ospedale Maggiore - University A. Avogadro of Novara (F. Della Corte); Osp. Molinette of Torino (P. P. Donadio); A.O. Umberto I Ancona, Rianimazione Clinica (A. Donati); Azienda Ospedaliera Universitaria Policlinico of Palermo (A. Giarratano); San Giovanni Di Dio of Florence (T. Giorgio); H San Raffaele IRCCS of Milano (D. Giudici); Ospedale Di Busto Arsizio (S. Greco); Civile Di Massa (A. Guadagnucci); San Paolo of Milano (G. Lapichino); S.Giovanni Bosco Torino (S. Livigni); Osp. San Giovanni of Sesto (G. Moise); S Camillo of Roma (G. Nardi); Vittorio Emanuele of Catania (E. Panascia); Hospital of Piacenza (M. Pizzamiglio); Universita di Torino-Ospedale S. Giovanni Battista (V. M. Ranieri); Policlinico Le Scotte of Siena (R. Rosi); Ospedale Maggiore Policlinico IRCCS of Milano (A. Sicignano); A. Uboldo of Cernusco Sul Naviglio (M. Solca); P.O. Civile Carrara of Massa (G. Vignali); San Giovanni of Roma (I. Volpe Rinonapoli); *Netherlands:* Boven IJ Ziekenhuis of Amsterdam (M. Barnas); UMC St Radboud of Nijmegen (E.E. De Bel); Academic Medical Center of Amsterdam (A-C. De Pont); VUMC of Amsterdam (J. Groeneveld); Groningen University Hospital (M Nijsten); Waterlandziekenhuis of Purmerend (L Sie); OLVG of Amsterdam (D. F. Zandstra); *Norway:* Sentralsjukehuset i Rogaland of Stavanger (S. Harboe); Sykehuset Østfold of Fredrikstad (S. Lindén); Aker University Hospital of Oslo (R. Z. Lovstad); Ulleval University Hospitalof Oslo (H. Moen); Akershus University Hospital of Nordbyhagen (N. Smith-Erichsen); *Poland:* Paediatric University Hospital of Lodz (A. Piotrowski); Central Clinic Hospital SLAM of Katowice (E. Karpel); *Portugal :* Garcia de Orta of Almada (E. Almeida); Hospital de St. António dos Capuchos of Lisboa (R. Moreno); Hospital de Santa Maria of Lisboa (A. Pais-De-Lacerda); Hospital S.Joao of Porto (J. A. Paiva); Fernado Fonseca of Masama (I. Serra); São Teotonio Viseu (A. Pimentel); *Romania:* Inst of Cardiovascular Diseases of Bucharest (D. Filipescu); *Serbia and Montenegro:* Military Medical Academy of Belgrade (K. Jovanovic); *Slovakia:* SUSCH of Bratislava (P. Malik); *Slovenia:* General Hospital of Novo Mesto (K. Lucka); General Hospital of Celje (G. Voga); *Spain:* Hospital Universitario Rio Hortega of Valladolid (C. Aldecoa Alvarez-Santullano); Sabadell Hospital (A. Artigas); Hospital Clinic of Barcelona (E. Zavala, A. Escorsell, J. Nicolas); Virgen del Camino of Pamplona (J. J. Izura Cea); Virgen de la Salud of Toledo (L. Marina); 12 de Octubre of Madrid (J. Montejo); Gregorio Maranon of Madrid (E. Palencia); General Universitario de Elche (F. Santos); Puerta del Mar of Cadiz (R. Sierra-Camerino); Fundación Jiménez Díaz of Madrid (F. Sipmann); Hospital Clinic of Barcelona (E. Zavala); *Sweden:* Central Hospital of Kristianstad (K. Brodersen); Stockholm Soder Hospital (J. Haggqvist); Sunderby Hospital of Luleå (D. Hermansson); Huddinge University Hospital of Stockholm (H. Hjelmqvist); *Switzerland:* Kantonsspital Luzern (K. Heer); Hirslanden Klinik Beau-Site of Bern (G. Loderer); University Hospital of Zurich (M. Maggiorini); Hôpital de la ville of La Chaux-de-Fonds (H. Zender); *United Kingdom:* Edinburgh Western General Hospital (P. Andrews); Peterborough Hospitals NHS Trust of Peterborough (B. Appadu); University Hospital Lewisham, London (C. Barrera Groba); Bristol Royal Infirmary (J. Bewley); Queen Elizabeth Hospital Kings Lynn (K. Burchett); Milton Keynes General (P. Chambers); Homerton University Hospital of London (J. Coakley); Charing Cross Hospital of London (D. Doberenz); North Staffordshire Hospital of Stoke On Trent (N. Eastwood); Antrim Area Hospital (A. Ferguson); Royal Berkshire Hospital of Reading (J. Fielden); The James Cook University Hospital of Middlesbrough (J. Gedney); Addenbrookes of Cambridge (K. Gunning); Rotherham DGH (D. Harling); St.Helier of Carshalton (S. Jankowski); Southport & Formby (D. Jayson); Freeman of Newcastle Upon Tyne (A. Kilner); University Hospital of North Tees at Stockton on Tees (V. Krishna-Kumar); St. Thomas Hospital of London (K. Lei); Royal Infirmary of Edinburgh (S. Mackenzie); Derriford of Plymouth (P. Macnaughton); Royal Liverpool University Hospital (G. Marx); Stirling Royal Infirmary (C. McCulloch); University Hospital of Wales, Cardiff (P. Morgan); St George's Hospital of London (A. Rhodes); Gloucestershire Royal Hospital (C. Roberts); St Peters of Chertsey (M. Russell); James Paget Hospital of Great Yarmouth (D. Tupper-Carey, M. Wright); Kettering General Hospital (L. Twohey); Burnley DGH (J. Watts); Northampton General Hospital (R. Webster); Dumfries Royal Infirmary (D. Williams)
